# Sec62 promotes stemness and chemoresistance of human colorectal cancer through activating Wnt/β-catenin pathway

**DOI:** 10.1186/s13046-021-01934-6

**Published:** 2021-04-15

**Authors:** Xiaofeng Liu, Kunqi Su, Xiaoyan Sun, Yang Jiang, Lijun Wang, Chenyu Hu, Chunfeng Zhang, Min Lu, Xiaojuan Du, Baocai Xing

**Affiliations:** 1 Hepatopancreatobiliary Surgery Department I, Key laboratory of Carcinogenesis and Translational Research (Ministry of Education/Beijing), Peking University Cancer Hospital & Institute, Beijing, 100142 China; 2 Department of Cell Biology, School of Basic Medical Sciences, Peking University Health Science Center, Beijing, 100191 China; 3 Department of Medical Genetics, School of Basic Medical Sciences, Peking University Health Science Center, Beijing, 100191 China; 4 Department of Pathology, School of Basic Medical Sciences, Peking University Health Science Center, Beijing, 100191 China

**Keywords:** Sec62, Chemosensitivity, Cancer stem cell, CRC, Wnt/β-catenin signaling, m^6^A modification

## Abstract

**Background:**

Cancer stem cell (CSC)-related chemoresistance leads to poor outcome of the patients with colorectal cancer (CRC). In this study, we identified the chemoresistance-relevant molecules and decipher the involved mechanisms to provide potential therapeutic target for CRC. We focused on Sec62, a novel target with significantly increased expression in chemoresistant CRC tissues, and further investigated its role in the progression of CRC.

**Methods:**

Through analyzing the differentially-expressed genes between chemoresistant and chemosensitive CRCs, we selected Sec62 as a novel chemoresistance-related target in CRC. The expression and clinical significance of Sec62 were determined by immunoblotting and immunohistochemistry in tissues and cell lines of CRC. The roles of Sec62 in drug resistance, stemness and tumorigenesis were evaluated in vitro and in vivo using functional experiments. GST pull-down, western blot, coimmunoprecipitation and Me-RIP assays were performed to further explore the downstream molecular mechanisms.

**Results:**

Sec62 upregulation was associated with the chemoresistance of CRC and poor outcome of CRC patients. Depletion of Sec62 sensitized CRC cells to chemotherapeutic drugs. Sec62 promoted the stemness of CRC cells through activating Wnt/β-catenin signaling. Mechanistically, Sec62 bound to β-catenin and inhibited the degradation of β-catenin. Sec62 competitively disrupted the interaction between β-catenin and APC to inhibit the β-catenin destruction complex assembly. Moreover, Sec62 expression was upregulated by the m^6^A-mediated stabilization of Sec62 mRNA.

**Conclusions:**

Sec62 upregulated by the METTL3-mediated m^6^A modification promotes the stemness and chemoresistance of CRC by binding to β-catenin and enhancing Wnt signalling. Thus, m^6^A modification-Sec62-β-catenin molecular axis might act as therapeutic targets in improving treatment of CRC.

**Supplementary Information:**

The online version contains supplementary material available at 10.1186/s13046-021-01934-6.

## Background

Colorectal cancer (CRC) is the fourth leading cause of cancer-related mortality [1]. Surgery is the cornerstone of treatment for CRC while adjuvant chemotherapy is routinely applied to improve the prognosis of the patients [2]. However, chemoresistance is one of the major problems hindering the CRC treatment [3]. Since the existence of cancer stem cells (CSCs) leads to chemotherapy failure and tumor recurrence, targeting the CSCs could improve the therapeutic effectiveness in CRC [4–6]. Thus, exploration of molecules controlling the stemness of CRC will provide therapeutic targets for CRC.

Wnt/β-catenin signaling plays crucial roles in the maintenance of CSCs [7]. The turnover of β-catenin is the critical event in Wnt/β-catenin signaling pathway [8]. Accumulated β-catenin enters the nucleus and binds to TCF transcription factors to activate the transcription of downstream genes including CD44, MYC and LGR5, which potentiate the stemness of CRC and promotes CRC progression [9–11]. Degradation of β-catenin is mediated by the β-catenin destruction complex, in which APC plays critical roles as it binds to AXIN1 and β-catenin [12]. In the majority (about 75%) of CRC, *APC* gene is mutated and produces the N-terminal truncated APC lacking of APC-AXIN1 interaction. Thus, the regulation of the APC-β-catenin interaction is critical in the activation of β-catenin in the CRC cells [13, 14]. However, the mechanisms by which the APC-β-catenin interaction is regulated in CRC are not fully understood.

Here, we screened the chemosensitivity-related genes and identified Sec62 as a chemoresistant target. Sec62 was originally found to locate in the membrane of endoplasmic reticulum (ER) [15]. Recent studies have uncovered that Sec62 is upregulated in various human cancers [16–18]. However, it’s unknown whether and how Sec62 acts in the CRC tumorigenesis and progression.

In the present study, we firstly evaluated the effect of Sec62 on the chemosensitivity and stemness of CRC. We further demonstrated that Sec62 activates β-catenin signaling to potentiate the stemness and attenuate the chemosensitivity in CRC. Additionally, Sec62 is upregulated by the METTL3-mediated m^6^A modification of Sec62 mRNA in CRC.

## Materials and methods

### Cell culture, antibodies and reagents

CRC cell lines were obtained from the Cell Resource Center (National Infrastructure of Cell Line Resource, NSTI) and cultured in DMEM or RPMI 1640 medium. All medium were supplemented with 10% fetal bovine serum. Cells were maintained in 5% CO_2_ at 37 °C in incubators with 100% humidity. Cell line authentications were performed by the provider. The antibodies and reagents for this study are listed in supplementary Table S1.

### Patient samples and tissue microarrays

Tissue microarrays consist of 102 formalin-fixed, paraffin-embedded CRC tissues and non-tumorous colorectal tissues obtained from the CRC patients who underwent curative surgical resection without prior neoadjuvant therapy from January 2004 to December 2008 in Peking University Cancer Hospital. The clinical pathologic characteristics of patients including age, gender, tumor location, carcinoembryonic antigen (CEA) level, tumor size, clinical stage, and distant metastasis are summarized in Supplemental Table S2.

### Cell transfection

Cells were transfected with plasmid DNA or siRNA RNA duplexes by Lipofectamine 2000 (Invitrogen) according to the manufacturer’s protocol. In transient transfection experiments, plasmid DNA was kept constant with empty vector. shRNAs were delivered by lentiviral infection with lentiviruses produced by transfection of HEK293T cells with the vector pLKO.1. Cells infected with lentiviruses delivering scrambled shRNA (shCtrl) were used as negative control cells. Short interfering RNA (siRNA) sequences were directly synthesized (GenePharma, Shanghai, China). The sequences of shRNAs and siRNAs are listed in the Table S3.

### Sphere formation assay

For sphere formation assay, a total of 800 cells were suspended in a serum-free medium and were plated into an ultralow attachment plate. Then, the cells were cultured in DMEM/F12 medium (Invitrogen) supplemented with insulin (Sigma), B27 (GIBCO), EGF (Sigma) and basic FGF (Sigma). For the serial passaging, the primary spheres were collected and resuspended in DMEM/F12 medium with the above supplements after trypsin dissociation. Finally, the number of spheres was counted under microscope and the size of spheres was estimated using Image J software.

### MTT assay and colony formation

For MTT assay, cells were seeded in a 96-well plate and cultured with indicated drugs for 72 h. Then, MTT assay was employed to assess cell viability according to the manufacturer’s protocol (Promega). For colony formation, cells were treated with DMSO or chemotherapeutic agents for 24 h, and subsequently seeded into 6-well plate (500 cell per well). After cultured for 12 days, the colonies were fixed with 4% paraformaldehyde and stained with 0.5% crystal violet. The visible colonies were counted and summarized.

### Flow cytometric analysis

Apoptosis assay was performed using Annexin V-FITC and propidium iodide staining kit (Keygen, Nanjing, China) according to the manufacturer’s protocol. To detect CD133^+^/CD44^+^ cells, CD133-PE (#130–090-853) and CD44-APC (#130–098-110) antibodies (Miltenyi Biotec.) were utilized to label cells. Then, labelled cells were subjected to flow cytometric analyses.

### In vivo chemo-resistance assay

DLD-1 cells were subcutaneously implanted into 4–6 weeks old female nude mice. When tumors reached a size of about 50 mm^3^, the nude mice were randomly divided into 6 groups. Group 1, 3 and 5 received an intratumoral injection of lentivirial-ctrl shRNA once per week for 5 weeks. Group 2, 4 and 6 received an intratumoral injection of lentivirial-Sec62 shRNA once per week for 5 weeks. At the same time, group 3 and 4 received an additional intraperitoneal injection of 5-Fu (0.15 mg/kg) twice per week for 4 weeks. Group 5 and 6 received an additional intraperitoneal injection of oxaliplatin (30 mg/kg) twice per week for 5 weeks. The tumor size was measured every 5 days and tumor volume was calculated using the formula V = 0.5 × W^2^ × L (V, volume; L, Length; W, Width).

### Coimmunoprecipitation assay

Cells were harvested and cell lysates were prepared in Buffer A (25 mM Tris-Cl pH 7.5, 150 mM KCl, 1 mM DTT, 2 mM EDTA, 0.5 mM PMSF, and 0.2% Nonidet P-40) and used for immunoprecipitation. The indicated antibodies were coupled with a 50% suspension of protein A-Sepharose beads (GE Healthcare) in Buffer IPP500 (500 mM NaCl, 10 mM Tris-Cl pH 8.0, 0.2% Nonidet P-40). Coupled beads were incubated with cell lysates for 2 h at 4 °C. After washing, the precipitates were examined by Western blot using the indicated antibodies.

### RNA immunoprecipitation (RIP)

RIP assay was carried out as previously described [19, 20]. Briefly, cells were treated with UV irradiation and lysed in high salt lysis buffer. Then, magnetic beads coated with 5 μg of antibodies were incubated with the prepared cell lysates overnight at 4 °C. The RNA-protein complexes were washed for 6 times and incubated with proteinase K digestion buffer. RNA was extracted by phenol-chloroform RNA extraction methods. The relative expression of RNA was determined by RT-qPCR and normalized to the input.

### In vivo limiting dilution assay

To investigate the effect of Sec62 on tumor self-renewal, an in vivo limiting dilution assay was performed. Female BABL/c nude mice (4 to 5 weeks old) were randomly divided into 4 groups (5 mice per group). Sec62-knockdown or control DLD1 cells were injected subcutaneously in the flank with a serial dilution of cells. At the end of 80 days, the tumor incidence of each group was observed and the stem cell frequency was estimated using an online tool at http://bioinf.wehi.edu.au/software/elda.

### Me-RIP (m^6^A-RNA immunoprecipitation) assay

Me-RIP assay was performed following published protocols [20, 21]. Briefly, Poly (A) mRNAs were isolated from total RNA with poly-T oligo attached magnetic beads (Promega). The cleaved RNA fragments were incubated for 2 h at 4 °C with m^6^A-specific antibody (No. 202003, Synaptic Systems, Germany) in IP buffer (50 mM Tris-HCl, 750 mM NaCl and 0.5% Igepal CA-630) supplemented with BSA. The mixture was then incubated with protein-A beads and eluted with elution buffer (1 × IP buffer and 6.7 mM m^6^A). Eluted RNA was precipitated by 75% ethanol. RNA was finally extracted by phenol-chloroform RNA extraction methods. The relative expression of RNA was assessed by qPCR and normalized to the input.

### Immunohistochemistry

Tissue sections were deparaffinized in xylene and rehydrated in ethanol. Then, the sections were treated with peroxidase solution and citrate buffer. After treatment with blocking buffer, sections were incubated with a primary antibody at 4 °C overnight. Next, the sections were visualized using an UltraVision Quanto Detection System HRP DAB Kit (ZSGB-Bio, China) according to the manufacturer’s protocols. The staining intensity were evaluated independently by two observers blinded to the clinical outcome. The percentages of positive tumor cells were scored as follows: 1%, 0 points; 1–25%, 1 point; 26–50%, 2 points; 51–75%, 3 points; and 75%, 4 points. The staining intensity was scored as follows: no staining, 0 points; weak staining, 1 point; moderate staining, 2 points; and strong staining, 3 points. Then, the two scores were multiplied to acquire a combined score ranging from 0 to 12.

### Immunofluorescence staining

Cells were fixed with 4% paraformaldehyde for 15 min and permeabilized using 0.2% Triton X-100 for 10 min at room temperature. After blocking with 10% goat serum, the cells were incubated with primary antibodies overnight at 4 °C. After washing with PBS, a FITC-conjugated anti-rabbit antibody and a TRITC-conjugated anti-mouse antibody were added, and the samples were incubated for 1 h at room temperature. Finally, the cells were stained with DAPI to visualize the nuclei.

### Statistical analyses

The significance of the differences was determined via one-way ANOVA or Student’s t-test. Spearman’s correlation coefficient was used to calculate the correlations between the two groups. Kaplan-Meier analysis was employed for survival analysis and the differences in the survival probabilities were estimated using the log-rank test. The statistical analyses were performed using GraphPad Prism or SPSS version 17.0 (SPSS, Inc.). All data was presented as mean ± SEM. A two-sided *P* < 0.05 was considered to indicate statistical significance. *P < 0.05 and **P < 0.01 for all the analyses.

## Results

### Depletion of Sec62 sensitizes CRC cells to chemotherapeutic drugs

**Fig. 1:**
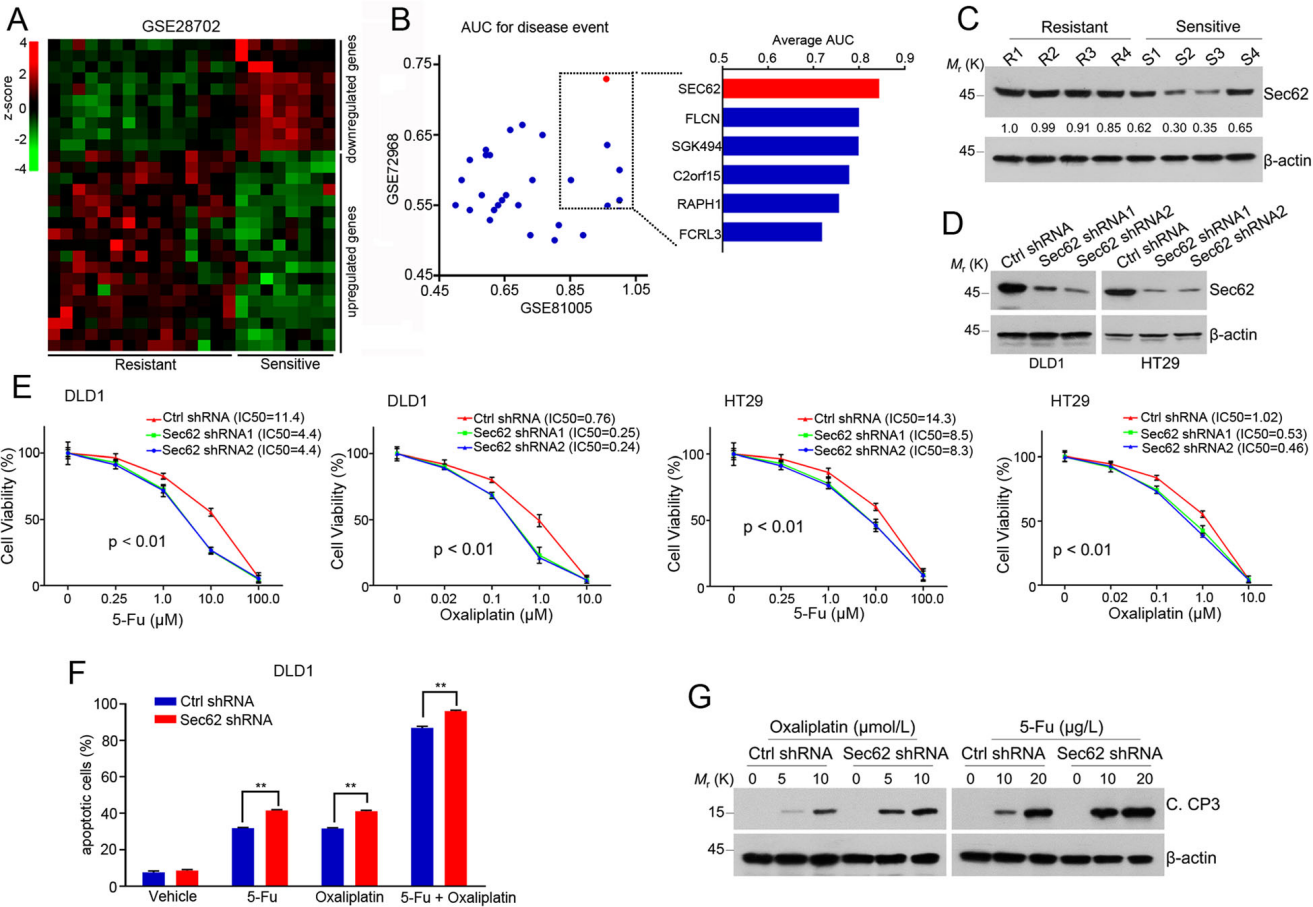
Depletion of Sec62 sensitizes CRC cells to chemotherapeutic drugs. **a** Heatmap showing DEGs in chemoresistant CRCs and chemosensitive CRCs based on dataset GSE28702. **b** AUC evaluation of prognostic value of candidate genes involved in chemoresistance-related events based on datasets GSE72968 and GSE81005. **c** The protein level of Sec62 was assessed by Western blot in chemoresistant CRC tissues (R1-R4) and chemosensitive CRC tissues (S1-S4). **d** Western blot was performed to evaluate Sec62 protein levels in Sec62-depleted DLD1 or HT29 cells. **e** The cells described in (**d**) were treated with an increasing concentrations of 5-Fu or oxaliplatin. After 72 h, the viable cells were determined by MTT assay. **f** The stably transfected cells were treated with 5-Fu or oxaliplatin for 24 h as indicated. The apoptotic cells were determined by flow cytometry using FITC-labeled Annexin V staining. **g** The stably transfected cells were treated with 5-Fu or oxaliplatin for 48 h. Whole-cell lysates were subjected to Western blot analyses using anti-cleaved caspase 3 (C. CP 3) antibody. β-actin was used as a loading control

To explore novel targets in chemosensitivity, we analyzed the DEGs (differentially-expressed genes) in two CRC cohorts consisting of chemoresistant and chemosensitive CRCs in dataset GSE28702. Eighteen genes were upregulated and 10 genes were downregulated in chemoresistant CRCs (Fig. 1a). AUC (area under curve) analysis were further performed to evaluate the involvement of candidate genes in CRC chemosensitivity using two independent datasets, GSE72968 and GSE81005 (Fig. 1b). Among these DEGs, the most significantly upregulated gene *SEC62* is of particular interest since its upregulation has been found to be associated with tumorigenesis [16] and the roles of Sec62 in CRC have not yet been illustrated. We evaluated the Sec62 expression levels in human CRC tissues and show that Sec62 expression was higher in the chemoresistant CRC tissues than that in the chemosensitive ones (Fig. 1c), suggesting that Sec62 might act in the CRC chemoresistance.

Next, we depleted Sec62 with two independent shRNAs (shRNA1 and shRNA2) in CRC cell lines and evaluated response of these cells to 5-Fu (fluorouracil) or oxaliplatin. Depletion of Sec62 sensitized the CRC cells to 5-Fu or oxaliplatin treatment ((Fig. 1d and e). Additionally, Sec62 depletion results in the increase of apoptotic cells in response to chemotherapeutic drugs (Fig. 1f). Consistently, the cleavage of Caspase 3 increased in Sec62-depleted cells under the drug treatment (Fig. 1g), confirming that depletion of Sec62 sensitizes CRC cells to the drug treatment. These data suggest that Sec62 suppresses the chemosensitivity of CRC cells.

### Sec62 maintains the stemness of CRC cells

Since chemoresistance is a key feature of cancer stemness [22], we wanted to know if Sec62 modulates the stemness of CRC cells. Linear regression analyses using The Cancer Genome Atlas (TCGA) CRC dataset indicated that Sec62 mRNA level is positively correlated with the CSC markers/regulators including CD44, CD133, EPCAM, ITGB1 and BMI1 (Additional file: Figure S1A), indicating that Sec62 might regulate the stemness of CRC.

**Fig. 2:**
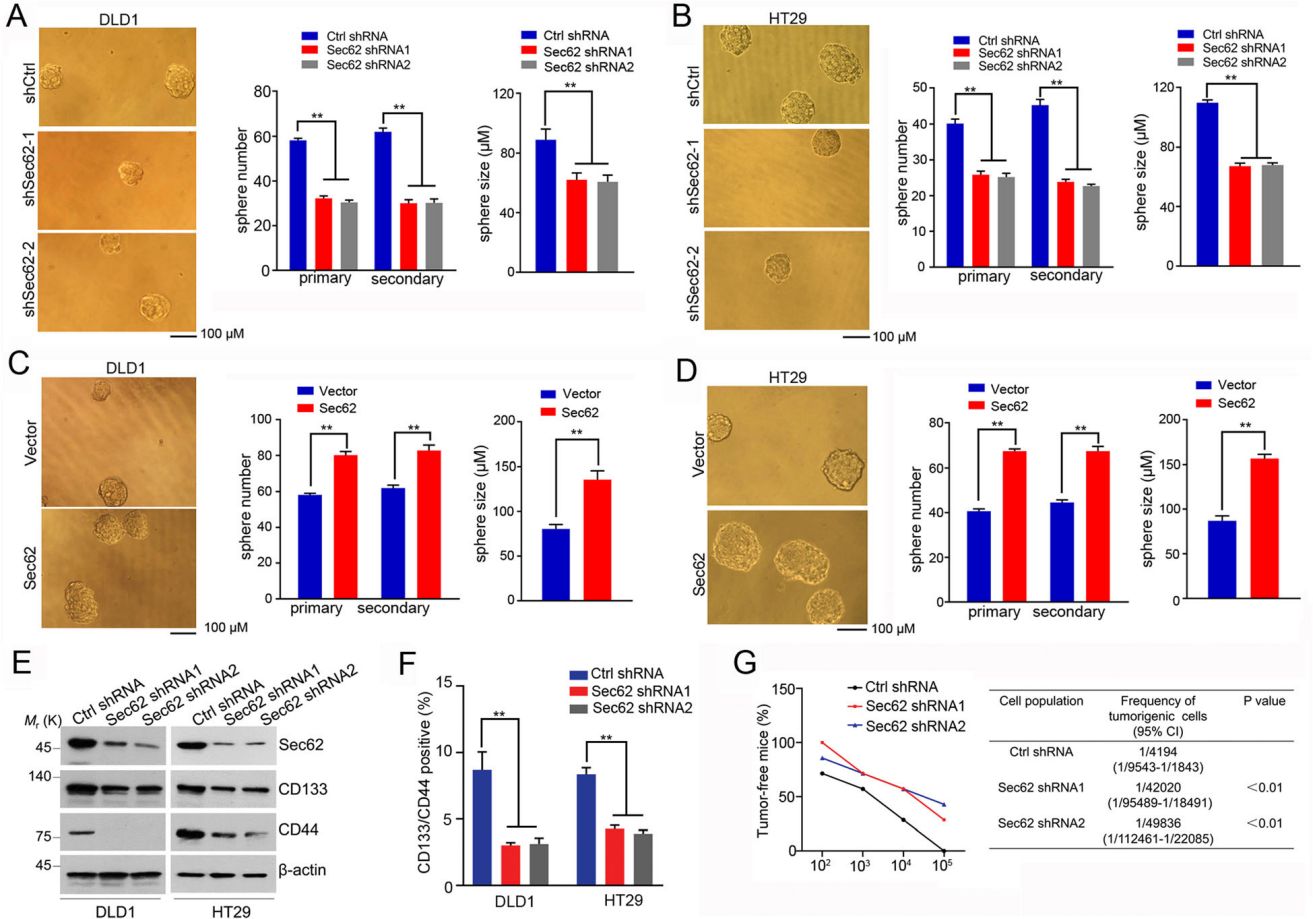
Sec62 promotes the stemness of CRC cells. **a**-**b** Spheres derived from DLD1 or HT29 cells treated as indicated (left). The summary of the number and size of spheres (right) (*n* = 5). **c**-**d** Spheres derived from DLD1 or HT29 cells stably expressing Sec62 or vector cells (left). The summary of the number and size of spheres (right) (*n* = 5). **e** Western blot analysis was performed to determine the expression of levels of indicated CSC markers in Sec62 knockdown and control cells. **f** CD133^+^/CD44^+^ cells were analyzed by flow cytometry in DLD1 or HT29 cells stably transcfected with Sec62 shRNA or control shRNA. **g** DLD1 Sec62-shRNA or DLD1 Ctrl shRNA cells were diluted and subcutaneously implanted into nude mice. Tumorigenic cell frequency was analyzed with a limiting dilution assay

To determine the effect of Sec62 on the stemness of CRC, tumor sphere assay was employed. The number and average diameter of the spheres derived from the Sec62-depleted CRC cells were less than those derived from the control cells (Fig. 2a, b and Additional file: Figure S1B), confirming that Sec62 enhances the stemness of CSCs. Accordingly, Sec62 overexpression enhanced primary and secondary spheroid formation (Fig. 2c, d and Additional file: Figure S1C). Importantly, two CSC markers CD133 and CD44 [23, 24], were downregulated in Sec62-depleted cells (Fig. 1e). Flow cytometry analysis also showed that the percentage of cells coexpressing CD44 and CD133 was reduced when Sec62 was depleted (Fig. 2f). These data revealed that Sec62 maintains the stemness of CRC. The limited dilution assay in mice xenografts showed that loss of Sec62 caused a great reduction of CSC frequency (Fig. 2g). The shSec62 xenografts displayed lower expression levels of CD44 and CD133 than shCtrl xenografts (Additional file: Figure S1D). These results further confirmed that Sec62 promotes the stemness of CRC.

### Sec62 interacts with β-catenin in CRC cells and in vitro

**Fig. 3:**
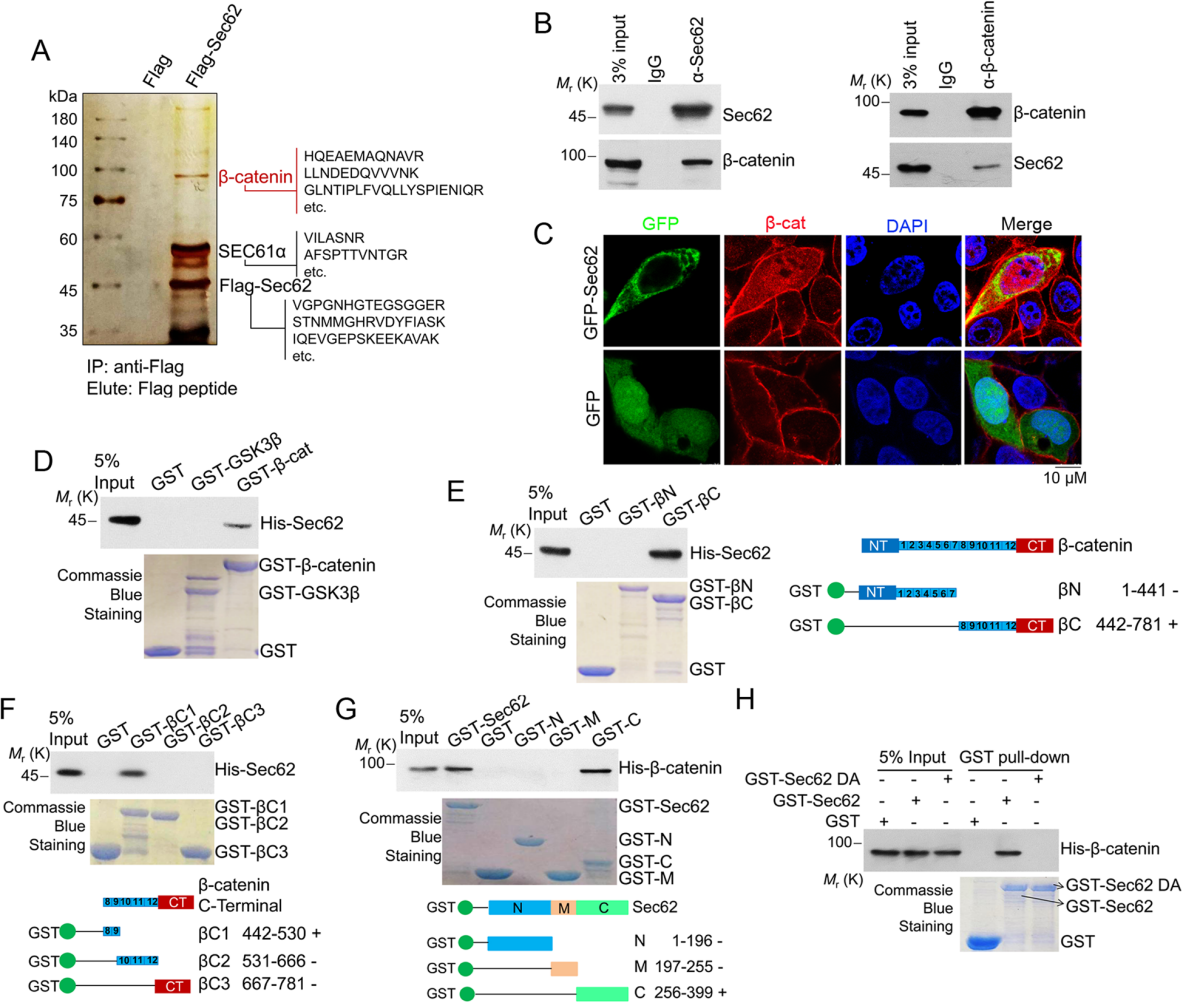
Sec62 interacts with β-catenin. **a** DLD1 cells were transfected with Flag-Sec62. The whole cell extracts were immunoprecipitated with an anti-Flag antibody affinity resin. Sec62-binding proteins were resolved by SDS-PAGE, detected by silver staining and analyzed by mass spectrometry. **b** DLD1 cell lysates were immunoprecipitated with anti-Sec62 antibody or anti-β-catenin antibody. Immunoprecipitants were immunoblotted with the indicated antibodies. **c** DLD1 cells were transfected with the indicated vectors and immunofluorescence was performed using anti-β-catenin antibody. **d** GST pull-down assay was performed with purified His-Sec62 and GST-β-catenin or GST-GSK3β proteins. The amounts of GST fusion proteins used in the GST pull-down assay are shown in the lower panel. **e** GST pull-down assay was performed with GST-β-catenin and His-Sec62 (left). Schematic diagram of β-catenin deletion mutants (right). **f** GST pull-down assays was performed with GST-β-catenin and His-Sec62 (upper). A schematic diagram of β-catenin deletion mutants is shown in the lower panel. The numbers in blue box indicate the ARM repeats. **g** GST pull-down was performed with GST-Sec62 and His-β-catenin (upper). Shown is a schematic diagram of GST-Sec62 deletion mutants (lower). **h** GST pull-down assays were performed with GST-Sec62 or GST-Sec62 DA

To uncover the mechanism by which Sec62 maintains the stemness in CRC cells, we purified the Flag-Sec62-specific immunocomplex from DLD1 cells and identified Flag-Sec62-interacting proteins by mass spectrometry (Fig. 3a). Among the Flag-Sec62-interacting proteins, β-catenin is of particular interest due to its pivotal role in cancer stemness [25].

The endogenous interaction between Sec62 and β-catenin was confirmed by co-immunoprecipitation (co-IP) (Fig. 3b). The co-localization of Sec62 and β-catenin was also observed in cells (Fig. 3c). The GST pull-down experiments performed with purified Sec62 and GST-β-catenin show that Sec62 specifically bound to GST-β-catenin other than GST-GSK3β (Fig. 3d), indicating that the Sec62 interacts with β-catenin in vitro. Further, the Sec62-binding region of β-catenin were narrowed down to the 8–9 arm repeats of armadillo (ARM) domain by GST pull-down experiments (Fig. 3e and f). The C-terminus of Sec62 was required for the interaction with β-catenin (Fig. 3g). Most of the β-catenin’s binding partners binds to β-catenin ARM repeats through the conserved DxqqxFx2-7E binding motifs (q and F denote hydrophobic and aromatic residues, respectively) [26]. We found a similar region in Sec62 C-terminus with high sequence consensus to the β-catenin binding motif and this β-catenin binding like (BCBL) motif is highly conserved in various species (Additional file: Figure S2A). We thus constructed a GST-Sec62 deletion mutant (GST-Sec62 DA) lacking the BCBL (Additional file: Figure S2B). Indeed, the GST-Sec62 DA muant lost the capability of binding to β-catenin (Fig. 3h). Together, these results indicate that Sec62 is a novel β-catenin binding protein.

### Sec62 stabilizes β-catenin in APC truncated CRC cells or Wnt-stimulated CRC cells carrying wild-type APC

**Fig. 4:**
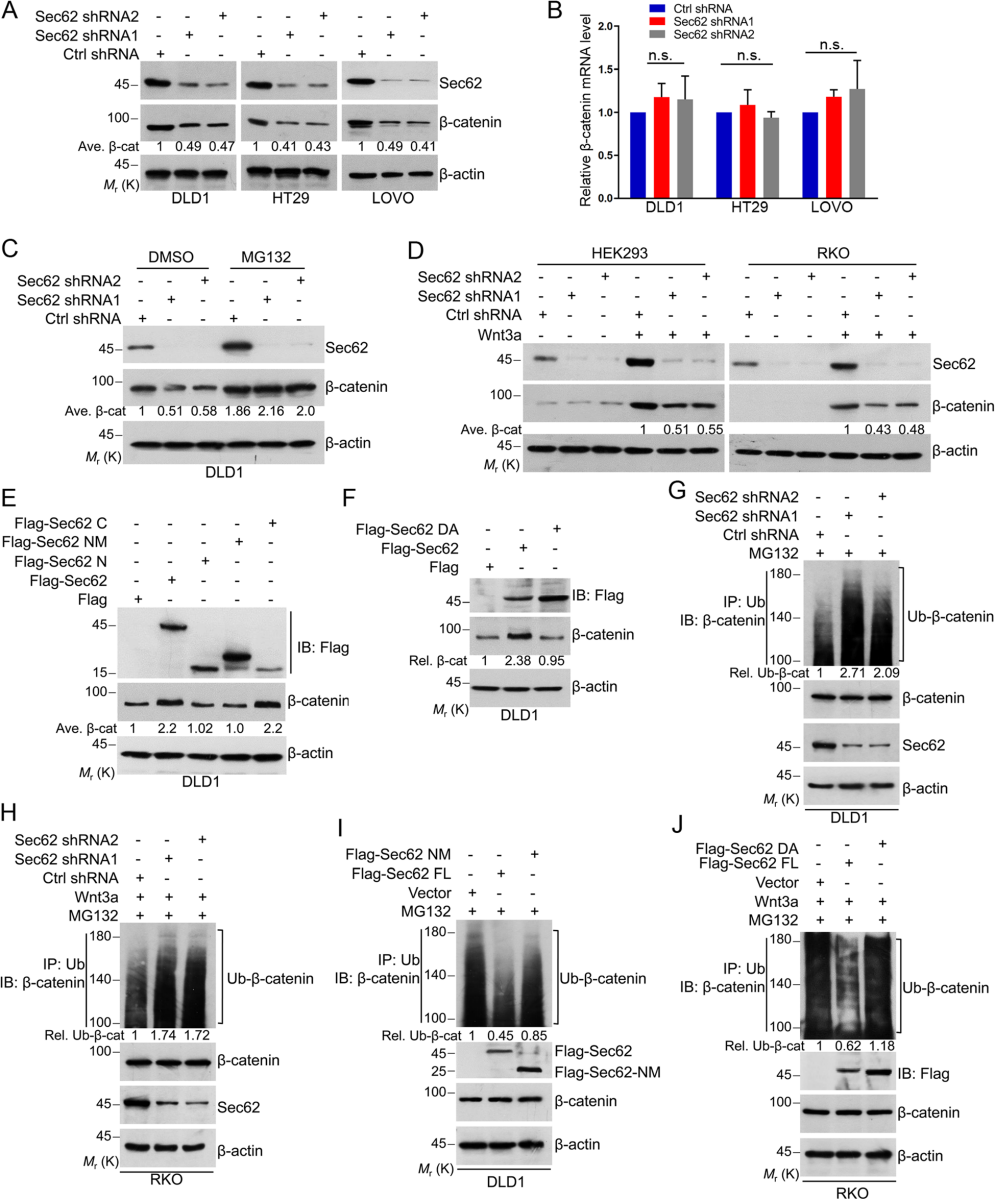
Sec62 stabilizes β-catenin through repressing its degradation. **a** Whole cell lysates extracted from the Sec62-depleted cells or control cells were subjected to Western blot using the indicated antibodies. **b** Total RNAs were extracted from Sec62-depleted and control cells and RT-qPCR were performed for evaluating the mRNA levels of β-catenin. **c** The Sec62-depleted and control cells were treated with MG132 and cell lystaes were subjected to Western blot performed with the indicated antibodies. **d** The Sec62-depleted and control shRNA cells were cultured in Wnt3a (0.2 μg/mL) contained medium for 6 h. Cell lysates were subjected to Western blot performed with the indicated antibodies. **e**-**f** DLD1 cells were transfected with Flag-Sec62 and its deletion mutants as indicated. Cells were harvested and cell lysates were subjected to Western blot using the indicated antibodies. **g** The Sec62-depleted and control cells were treated with MG132. The ubiquitination level of β-catenin was analyzed as indicated. **h** The Sec62-depleted and control RKO cells were treated with MG132. The ubiquitination level of β-catenin was analyzed as indicated. **i**-**j** DLD1 (**i**) or RKO (**j**) cells were transfected as indicated and treated with MG132. The ubiquitination level of β-catenin was analyzed as indicated

Knockdown of Sec62 significantly decreased the β-catenin protein level without affecting its mRNA level in DLD1, HT29 and LOVO cells (Fig. 4a and b). This effect was reversed by the proteasome inhibitor MG132 (Fig. 4c), suggesting that Sec62 depletion promotes proteasome-mediated β-catenin degradation. Since APC is truncated in these cells, we analyzed β-catenin level after depleting Sec62 in HEK293 and RKO cells which express wild-type (WT) APC with little autocrine Wnt signaling. Depletion of Sec62 reduced β-catenin levels only when these cells were treated by Wnt3a (Fig. 4d). These findings suggest that Sec62 inhibits β-catenin degradation in APC-truncated cells or in WT APC cells when Wnt signal is present. Further, we determined if Sec62 regulates β-catenin level dependent on the BCBL motif. Flag-Sec62 and Flag-Sec62 C-terminal increased β-catenin level, while the Flag-Sec62 NM or Flag-Sec62 DA mutants lost this capability (Fig. 4e and f), indicating that Sec62 controls β-catenin level through binding to β-catenin.

The ubiquitin-proteasome system is critical for determination of protein stability, including β-catenin stability [27]. Thus, we evaluated the effect of Sec62 intervention on ubiquitination of β-catenin. Sec62 depletion enhanced poly-ubiquitination of β-catenin in DLD1 cells (Fig. 4g). Knockdown of Sec62 increased poly-ubiquitination of β-catenin in RKO cells treated with Wn3a (Fig. 4h). Accordingly, Flag-Sec62, but not the Flag-Sec62 NM or DA mutant reduced the poly-ubiquitination of β-catenin in DLD1 or RKO cells (Fig. 4i and j). These results demonstrated that Sec62 inhibits poly-ubiquitination and degradation of β-catenin.

### Sec62 disrupts the activity of the β-catenin destruction complex

**Fig. 5:**
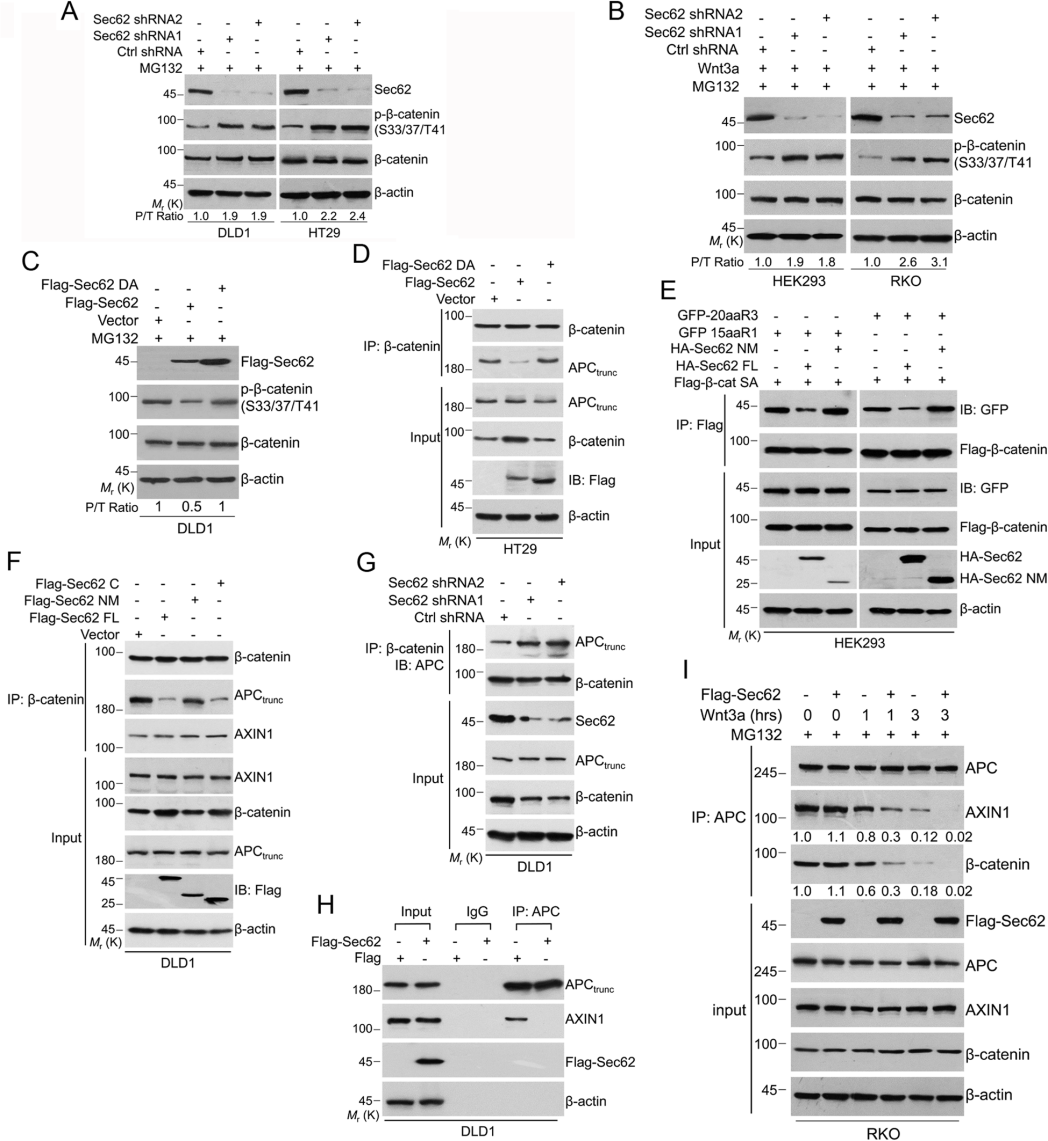
Sec62 disrupts the β-catenin destruction complex. **a** Phosphorylation level of β-catenin in Sec62-depleted CRC cells was analyzed by immunoblotting (left). The ratio of phosphorylated β-catenin/total β-catenin (P/T) is summarized (right) (*n* = 3). **b** Phosphorylation level of β-catenin in Sec62-depleted RKO or HEK293 cells was detected by immunoblotting after Wnt3a treatment (*n* = 3). **c** DLD1 cells were transfected with indicated plasmids and phosphorylation level of β-catenin was analyzed by western blot (*n* = 3). **d** HT29 cells were transfected with indicated plasmids and co-IP was performed with the cell lysates using anti-β-catenin antibody. Western blot was performed on the precipitates using the indicated antibodies. **e** HEK293 cells were transfected with indicated plasmids and cell lysates were subjected to co-IP using anti-Flag antibody. **f** DLD1 cells were transfected with indicated plasmids and cell lysates were subjected to co-IP using anti-β-catenin antibody. **g** The Sec62-depleted and control cells were harvested and cell lysates were subjected to co-IP using anti-β-catenin antibody. **h** DLD1 cells transfected with indicated plasmids and cell lysates were subjected to co-IP using anti-APC antibody. **i** RKO cells were transfected with Flag-Sec62 or Flag vetor and cultured with Wnt3a medium. The cell lysates were subjected to co-IP using anti-APC antibody

The activity of β-catenin destruction complex plays essential role in controlling β-catenin stability [8, 28]. To determine if Sec62 impairs the activity of β-catenin destruction complex, the ratio of phosphorylated β-catenin/total β-catenin was evaluated as previously described [29]. The ratio of phospho-β-catenin/total β-catenin was increased upon knockdown of Sec62 in DLD1 and HT29 cells (Fig. 5a). Moreover, Sec62 depletion increased the ratio of phospho-β-catenin/total β-catenin in Wnt3a-treated HEK293 or RKO cells (Fig. 5b). In contrast, Flag-Sec62 reduced the ratio of phospho-β-catenin/total β-catenin, while the Sec62 DA mutant lost this function (Fig. 5c). Furthermore, Sec62 depletion decreased the expression of AXIN2, one of β-catenin downstream genes, and had no effect on AXIN1 level, which participates in the assembly of the β-catenin destruction complex (Additional file: Figure S3A). Taken together, our results demonstrated that Sec62 inhibits the activity of the β-catenin destruction complex to enhance β-catenin activation.

### Sec62 inhibits APC-β-catenin interaction

Since Sec62 binds to the 8–9 ARM repeat domain of β-catenin which also interacts with APC, Sec62 might compete with APC for binding to β-catenin. Flag-Sec62, but neither the Flag-Sec62 DA nor Flag-Sec62 NM mutant, inhibited the interaction between APC and β-catenin (Fig. 5d and Additional file: Figure S3B, C), indicating that Sec62 disrupts the β-catenin-APC interaction through binding to β-catenin. Since the 15aa and 20aa repeats of APC binds to the ARM repeats 6–10 of β-catenin [30], we thus examined the interaction between GFP-APC 15aaR1 or GFP-APC 20aaR3 and β-catenin when HA-Sec62 was overexpressed. HA-Sec62, but not the HA-Sec62 NM mutant, blocked the interaction between β-catenin and GFP-APC 15aaR1 or GFP-APC 20aaR3 (Fig. 5e), confirming that Sec62 competes with APC for the binding to β-catenin. However, the interaction between β-catenin and AXIN1 was not affected by Flag-Sec62 (Fig. 5f). Moreover, the interaction between APC and β-catenin was enhanced by knockdown of Sec62 (Fig. 5g), indicating that endogenous Sec62 attenuates the interaction between APC and β-catenin.

Given that β-catenin bridges the interaction between the truncated APC and AXIN1 in the assembly of the β-catenin destruction complex [14], we wondered if Sec62 affects the APC-AXIN1 interaction. Flag-Sec62 reduced the APC-AXIN1 interaction in DLD1 cells (Fig. 5h). Thus, Sec62 inhibits the assembly of β-catenin destruction complex by binding to β-catenin in the APC-truncated CRC cells. Then, we evaluated the effect of Sec62 on the APC-AXIN1 interaction in the cells expressing WT APC. Wnt3a treatment resulted in the dissociation of APC with AXIN1 as previously reported [14] and Flag-Sec62 enhanced this effect, indicating that Sec62 reinforces the dissociation of APC-AXIN1 upon Wnt signal (Fig. 5i). Together, Sec62 protects β-catenin from degradation through inhibiting APC-β-catenin interaction.

### Sec62 promotes CRC progression by activating the β-catenin signaling

**Fig. 6:**
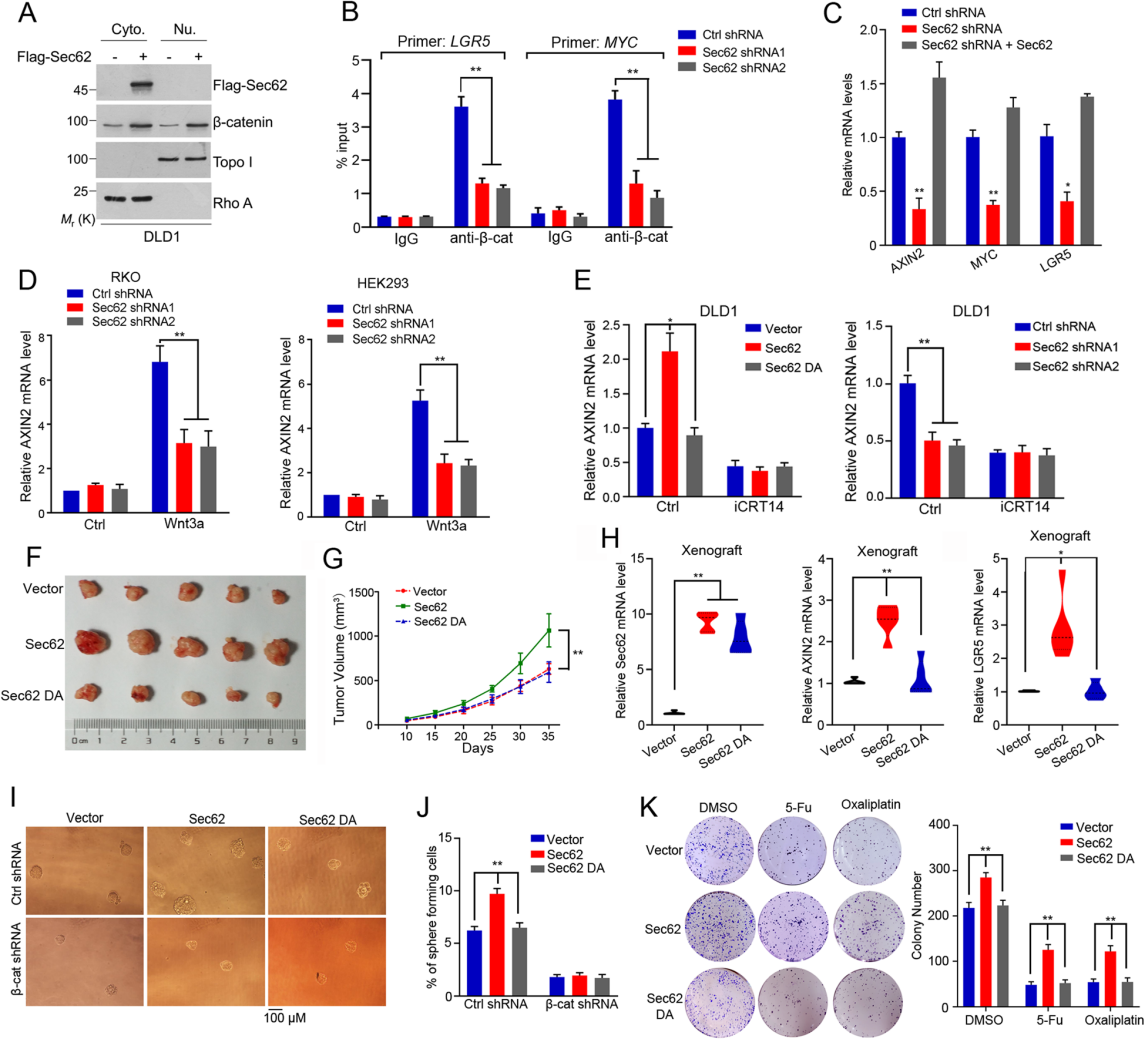
Sec62 promotes CRC progression by activating β-catenin signaling. **a** DLD1 cells were transfected with indicated vectors. Cell fraction was performed and the nuclear β-catenin was evaluated by Western blot. **b** DLD1 cells were treated as indicated. The promoter occupancy of *LGR5* or *MYC* by β-catenin was analyzed by ChIP assay. **c** The Sec62 depleted and control cells were transfected with Sec62 plasmid. RT-qPCR was performed to analyze the mRNA levels of the indicated genes. **d** The Sec62-depleted and control cells were treated with Wnt3a. Total RNAs were extracted and RT-qPCR was performed as indicated. **e** The Sec62-depleted and control cells were treated with iCRT14 (50 μM for 24 h). RT-qPCR was performed to analyze the mRNA levels of the indicated genes. **f** Tumor formation in nude mice injected with Sec62-overexpressed, Sec62 DA-overexpressed DLD-1 or control cells. **g** The tumor growth curves of each group of mice were summarized (*n* = 5). **h** Total RNAs were extracted from the xenografts obtained in (F). RT-qPCR was performed for evaluating the indicated genes (*n* = 5). **i**-**j** DLD1 cells were infected with indicated lentivirus and sphere formation assays were performed in sphere-formation medium (**i**). The number of spheres derived from indicated cells were summarized (**j**) (*n* = 6). **k** Colony number quantification of the DLD1 cells after treatment with 5-Fu or oxaliplatin (*n* = 6)

Based on the observed effect of Sec62 on β-catenin levels, we further investigated whether Sec62 regulated the nuclear translocation of β-catenin and activation of β-catenin signaling. Overexpression of Sec62 increased the nuclear accumulation of β-catenin (Fig. 6a). Moreover, Sec62 knockdown decreased β-catenin recruiment to the promoter of Wnt target genes, such as MYC and LGR5 (Fig. 6b). Accordingly, depletion of Sec62 decreased the expression of Wnt target genes including AXIN2, LGR5 and MYC (Fig. 6c), all of which play critical roles in maintaining cancer cell stemness and CRC progression [4, 31, 32]. To determine if Sec62 regulates the downstream gene of β-catenin in CRC cells expressing WT APC, we evaluated AXIN2 mRNA levels in RKO and HEK293 cells. Knockdown of Sec62 repressed the transcription of AXIN2 in the presence of Wnt3a in these cells (Fig. 6d). Moreover, Flag-Sec62, but not Flag-Sec62 DA mutant, upregulated AXIN2 mRNA levels and this effect was blocked by a specific β-catenin/TCF transcription inhibitor iCRT14 (Fig. 6e). Consistently, depletion of Sec62 downregulated the AXIN2 mRNA level and iCRT14 blocked this effect (Fig. 6e). Together, Sec62 promotes the β-catenin/TCF mediated transcription. We further examined if Sec62 activates β-catenin signaling dependent on BCBL motif in vivo. Overexpression of Sec62 promoted tumour growth in the mouse xenografts while Sec62 DA mutant failed to do so (Fig. 6f and g). Moreover, the mRNA levels of AXIN2 and LGR5 in the xenografts were upregulated by Sec62 but not by Sec62 DA mutant (Fig. 6h). Thus, Sec62 activates the Wnt/β-catenin signaling by binding to β-catenin.

Next, we assessed if Sec62 controls the stemness of CRC through activating β-catenin signalling. Overexpression of Sec62 increased the number of oncosphere while Sec62 DA mutant lost this capability (Fig. 6i and j). Further, overexpression of Sec62 failed to potentiate the stemness when β-catenin was depleted, demonstrating that Sec62 maintains the stemness of CRC dependent on β-catenin. Clony formation was decreased by 5-Fu or oxaliplatin and this effect was reversed by overexpression of Sec62 but not the Sec62 DA mutant, indicating that Sec62 attenuates the chemosensitivity of CRC cells through activating β-catenin (Fig. 6k). Collectively, we demonstrated that Sec62 maintains the stemness by activating the Wnt signalling pathway in CRC.

### Upregulation of Sec62 correlates with poor prognosis of CRC patients

**Fig. 7:**
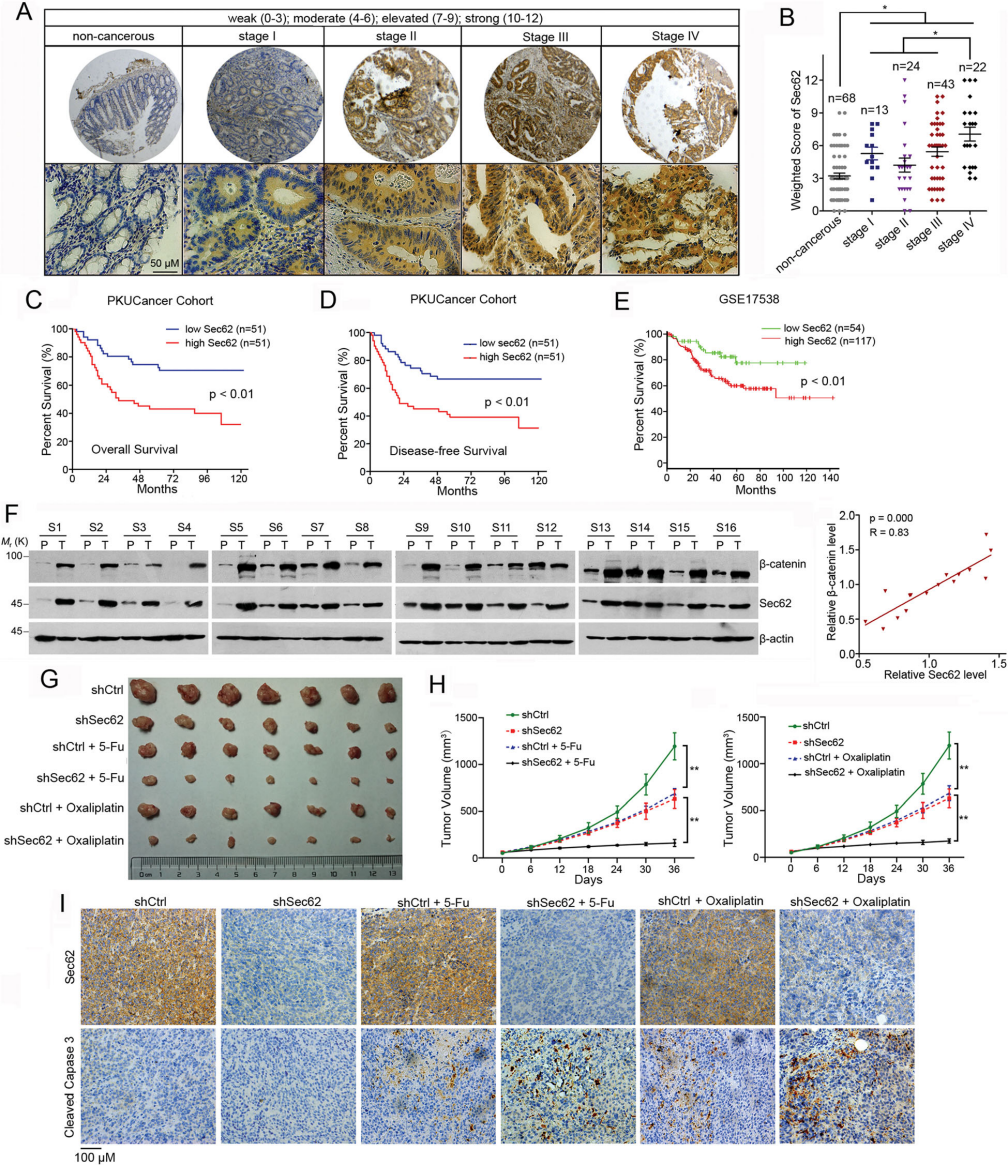
Sec62 correlates with poor prognosis of the CRC patients. **a** Representative immunostaining of Sec62 in CRC samples at stage I-IV and adjcent non-cancerous tissues. **b** The weighted scores of the IHC staining for Sec62 were plotted based on the clinical stages. **c**-**d** Kaplan-Meier survival curve for overall survival (**c**) and disease-free survival (**d**) of the CRC patients. Statistics were evaluated by the log-rank test. **e** The survival of the CRC patients with different Sec62 mRNA levels from GSE17538 were analyzed by Kaplan-Meier. **f** Western blot analysis of Sec62 and β-catenin expression in 16 individual paired CRC tissues. The correlation between Sec62 protein level and β-catenin protein level in the CRC tissues was plotted (Right, *n* = 16). **g** Images of the mouse xenograft tumors treated with Sec62 shRNA lentivirus (shSec62) or control shRNA lentivirus (shCtrl) with or without 5-Fu or oxaliplatin. **h** The tumor growth curves of the mouse xenografts (*n* = 7). **i** Sec62 and cleaved caspase 3 were evaluated by IHC in the mouse xenografts

We next determined the relationship between the Sec62 expression and prognosis of the CRC patients. Sec62 expression was evaluated by IHC in 102 cases of CRC samples. Sec62 was upregulated in the cancer tissues compared with non-cancerous tissues and significantly increased at clinical stage IV (Fig. 7a and b). Importantly, the high Sec62 expression was correlated with shorter overall survival and disease-free survival in the patients (Fig. 7c and d). The GSE17538 data also showed that high Sec62 expression predicts shorter survival in 271 cases of CRC patients (Fig. 7e). These findings indicate that Sec62 is upregulated in CRC and predicts poor prognosis of CRC patients. The expression of Sec62 and β-catenin on 16 paired cancerous and matched non-cancerous sections of human CRC tissues was evaluated by immunoblotting. Sec62 expression was significantly correlated with β-catenin in CRC tissues (Fig. 7f). Moreover, Sec62 expression was positively correlated with the expression of β-catenin downstream genes, including CD44 and MYC, in CRC tissues (Additional file: Figure S4A), indicating that Sec62 activates β-catenin in CRC tissues.

We further explored if Sec62 affects the chemosensitivity of CRC cells in vivo. The nude mice bearing tumor xenograft were injected with control lentivirus (shCtrl) or Sec62 shRNA (shSec62)-lentivirus. Meanwhile, 5-Fu or oxaliplatin was injected. The results showed that Sec62 knockdown effectively attenuated tumor growth as 5-Fu or oxaliplatin did (Fig. 7g and h). Tumors treated with Sec62-shRNA lentivirus were more sensitive to 5-Fu or oxaliplatin when compared with those treated with control-shRNA lentivirus, indicating that Sec62 suppresses the chemosensitivity of CRC cells. IHC confirmed that the expression of Sec62 was inhibited by lentiviral-shSec62 in xenografts (Fig. 7i). Accordingly, Sec62 depletion caused more apoptotic cells upon 5-Fu or oxaliplatin treatment (Fig. 7i). The shSec62 xenografts displayed lower expression levels of CD44 and c-Myc than shCtrl xenografts, indicating that Sec62 promoted the activation of β-catenin (Additional file: Figure S4B). These data demonstrated that depletion of Sec62 sensitized CRC cells to chemotherapeutics in vivo.

### Sec62 expression is regulated by the METTL3-induced m^6^A modification

**Fig. 8:**
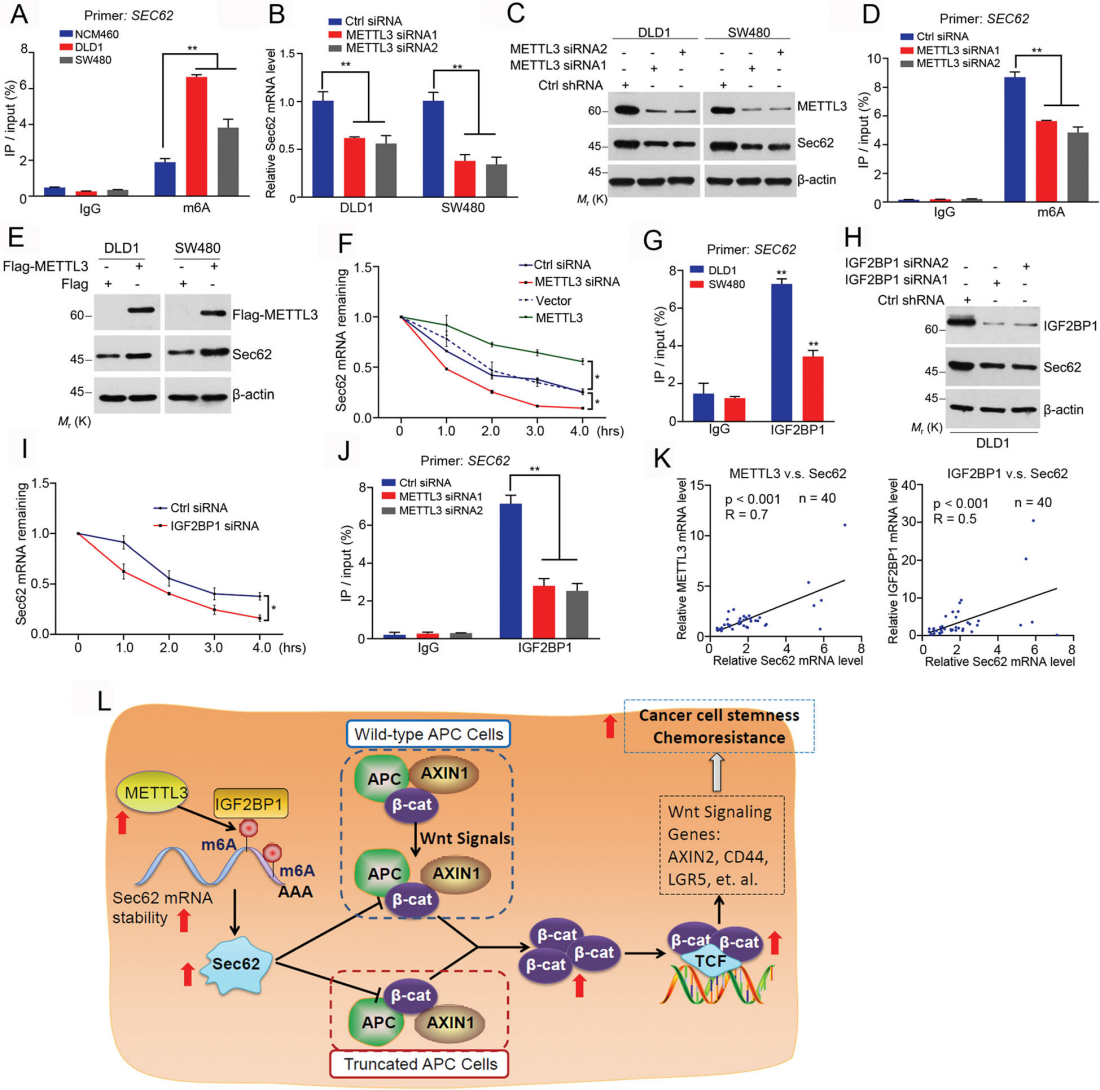
Sec62 expression is upregulated by the METTL3-induced m^6^A modification. **a** Anti-m^6^A IP was performed with cell lysates of NCM460, DLD1 or SW480 cells using anti-m^6^A antibody. RT-qPCR was performed to detect Sec62 mRNA in the elutes. **b** DLD1 or SW480 cells were transfected with the indicated siRNAs. RT-qPCR was performed for evaluating the indicated genes. **c** DLD1 or SW480 cells were transfected with the indicated siRNAs. Western blot was performed using the indicated antibodies. **d** DLD1 cells were transfected with the indicated siRNAs. Me-RIP was performed using anti-m^6^A antibody in the cell lysates. RT-qPCR was performed to detect Sec62 mRNA in the elutes. **e** DLD1 or SW480 cells were transfected with the indicated plasmids. Western blot was performed using the indicated antibodies. **f** DLD1 cells were transfected with siRNAs or plasmids as indicated. Cells were treated with 2.5 μM actinomycin D and mRNA levels of Sec62 were analyzed by RT-qPCR (*n* = 3). **g** RIP experiments were performed with cell lysates from DLD1 or SW480 cells using anti-IGF2BP1 antibody. Coprecipitated RNA was purified and Sec62 mRNA was analyzed by RT-qPCR. **h** DLD1 cells were transfected with indicated siRNAs. Western blot was performed using the indicated antibodies. **i** DLD1 cells were transfected as indicated. After treatment with actinomycin D for indicated times, the decay of Sec62 mRNA was analyzed (*n* = 3). **j** DLD1 cells were transfected as indicated. RIP was performed and Sec62 mRNA level in the elutes was analyzed by RT-qPCR. **k** Total RNAs were extracted for CRC tissues and RT-qPCR was performed for the indicated genes. The correlations between Sec62 mRNA level and METTL3 mRNA level or IGF2BP1 mRNA level were plotted (*n* = 40). **l** A working model explaining how the m^6^A modification-mediated Sec62 induction promotes stemness of human colorectal cancer cell

Finally, we explored how Sec62 is overexpressed in cancers. Since both the mRNA and protein levels of Sec62 are upregulated in CRC tissues, Sec62 expression might be regulated at mRNA level. Numerous m^6^A sites were found in Sec62 mRNA by RMBase prediction, suggesting that Sec62 mRNA might be controlled by m^6^A modification. We show that m^6^A was significantly more enriched in Sec62 mRNA in DLD1 or SW480 cells than that in normal colorectal cell NCM460 (Fig. 8a). We found that METTL3 depletion reduced Sec62 mRNA and protein levels (Fig. 8b and c). Importantly, depletion of METTL3 decreased m^6^A modification of Sec62 mRNA (Fig. 8d). Accordingly, Flag-METTL3 increased Sec62 protein level (Fig. 8e). These data revealed that Sec62 is upregulated by METTL3-induced m^6^A modification of Sec62 mRNA.

Since m^6^A modification controls mRNAs stability [33], we evaluated the Sec62 mRNA stability upon METTL3 knockdown. The Sec62 mRNA stability was decreased by METTL3 depletion (Fig. 8f), while it was increased by Flag-METTL3, indicating that METTL3 enhances Sec62 mRNA stability by controlling its m^6^A modification. It’s reported that IGF2BP1 (Insulin-like growth factor 2 mRNA-binding protein 1), a“reader” of m^6^A, plays a specific role in reading m^6^A and controlling the stability of m^6^A-modified mRNA [34]. We showed that IGF2BP1 interacted with Sec62 mRNA in CRC cells (Fig. 8g) and silencing IGF2BP1 decreased Sec62 expression levels (Fig. 8h). The half-life of Sec62 mRNA was reduced upon IGF2BP1 depletion (Fig. 8i). Finally, the interaction between IGF2BP1 and Sec62 mRNA was impaired after METTL3 depletion (Fig. 8j), indicating that IGF2BP1 binds to the m^6^A-modified Sec62 mRNA to maintain the Sec62 mRNA stability. Additionally, METTL3 or IGF2BP1 expression was positively correlated with Sec62 expression in CRC tissues (Fig. 8k). Together, we demonstrated that Sec62 is upregulated by m^6^A-mediated stabilization of Sec62 mRNA in CRC.

## Discussion

Chemosensitivity of CRC cells plays crucial roles in cancer treatment, which is tightly associated with cancer stem cells [22]. However, the mechanisms involved in the regulation of the colorectal cancer stem cells need to be clarified. In the present study, we set out to screen the chemosensitivity-related targets using GEO datasets [35–37], aiming to identify novel molecules regulating stemness and chemosensitivity in CRC. *SEC62* is one of the most significantly upregulated gene in chemoresistant CRCs in the screening. We further show that depletion of Sec62 sensitizes CRC cells to drug treatment and attenuates cancer stemness. Thus, inhibition of Sec62 function might be a potential therapeutic strategy to ameliorate chemoresistance in CRC.

Sec62 was initially found as a translocation-related protein in the membrane of endoplasmic reticulum [38, 39]. Recent studies have reported that Sec62 is correlated with metastasis in breast cancer and promotes recurrence of hepatocellular carcinoma, suggesting that Sec62 is associated with cancer progression [40, 41]. However, the mechanism by which Sec62 acts in the tumorigenesis remains unknown. In this study, we identified Sec62 as an important regulator of CRC stemness. Sec62 is upregulated in CRC and its overexpression is associated with poor prognosis of the patients. We further demonstrated that Sec62 promotes cancer stemness and CRC progression through enhancing Wnt/β-catenin signaling.

Activation of Wnt signaling leads to the stabilization of β-catenin to upregulate the transcription of its dowmstream genes [8]. β-catenin is abnormally activated in most of CRC patients, making it become a crucial therapeutic target [25, 42]. The accumulation of nuclear β-catenin enhances both chemoresistance and radioresistance in advanced rectal cancer [43]. Inhibitors of β-catenin signaling have been used to attenuate chemoresistance in CRC. For instance, the β-catenin inhibitor IC-2 increased the cytotoxicity of 5-Fu and downregulated the expression of CSC markers in CRC cells [44]. Zerumbone could suppress the stemness of CRC by inhibiting the β-catenin signaling [45]. Therefore, identification of molecules blocking the Wnt/β-catenin signaling would contribute to improve the chemotherapeutics in CRC. Here, we demonstrate that Sec62 enhances β-catenin stability and potentiates β-catenin signaling in CRC cells. Targeting Sec62 could improve the chemotherapeutic effect in Sec62-upregulated CRC patients by inhibiting β-catenin signaling.

Since the β-catenin destruction is the critical event in the Wnt/β-catenin signaling, the regulation of β-catenin stabilization will provide molecular evidence for understanding the activation of Wnt/β-catenin signaling. As a critical negative regulator of Wnt/β-catenin signaling, APC is essential for the assembly of the β-catenin destruction complex to destroy β-catenin. Since the SAMP repeats in APC are truncated leading to the loss of APC-AXIN1 interaction in 75% of CRC [13], the interaction between APC and β-catenin is critical for the assembly of the β-catenin destruction complex. Disruption of APC-β-catenin interaction often leads to hyperactivation of β-catenin in CRCs. However, the molelcular mechanism by which the APC-β-catenin is regulated remains unclear. APC binds to the ARM repeats 6–10 of β-catenin through its 15aa and 20aa repeats to facilitate the β-catenin degredation [14]. Here, we found that Sec62 binds to the ARM repeats 8–9 of β-catenin through the BCBL motif and inhibited the interaction between β-catenin and APC. Moreover, Sec62 disrupts the assembly of the β-catenin destruction complex and promotes β-catenin activation. Significantly, Sec62 maintains the stemness of CRC by enhancing the β-catenin signaling depending on the BCBL motif. Thus, we demonstrated that Sec62 activates β-catenin in the APC-truncated CRC. We further found that Sec62 activates β-catenin in the CRC cells expressing WT APC when Wnt signal is present. The Sec62-mediated β-catenin activation is verified by the co-upregulation of Sec62 and β-catenin in the CRC tissues. We thus provide Sec62 as a key activator of β-catenin in CRC.

Aberrant overexpression of Sec62 has been found in various tumors, but the underlying mechanism was unknown. Recently, accumulated studies show that m^6^A modification is one of important epigenetic regulatory mechanisms and is involved in pre-mRNA splicing, RNA stability and translation efficiency [46]. Dysregulated m^6^A modification of specific mRNAs is associated with cancer development and progression. As a m^6^A ‘wiriter’, METTL3 participates in the persisting stem-like phenotype and preventing the radiation-induced cytotoxicity through modulating mRNA stability such as SOX2 and CBX8 in the m^6^A dependent manner [20, 47]. Here, we show that m^6^A modification induced by METTL3 increased the stability of Sec62 mRNA, leading to upregulation of Sec62 in CRC. Thus, our findings provide *SEC62* as a new target gene regulated by METTL3 in the regulation of CRC cell stemness. The m^6^A “readers”, IGF2BP1, recognizes the consensus GG(m^6^A) C sequence and enhances the stability of targeted mRNAs in an m^6^A-dependent way [34]. In the present study, we found that IGF2BP1 binds to Sec62 mRNA and upregulates Sec62 expression in an m^6^A-dependent manner, which is consistent with previous findings.

## Conclusions

In summary, we show for the first time that Sec62 is commonly upregulated in CRC and its overexpression is associated with poor survival for patients with CRC. METTL3-mediated m^6^A modification of Sec62 mRNA upregulated Sec62 expression in CRC. Subsequently, Sec62 potentiates Wnt signaling through repressing β-catenin binding to APC complex, thus facilitates CRC cell stemness and chemoresistance (Fig. 7l). Therefore, Sec62 might be a potential target for CRC treatment.

## Supplementary Information

**Additional file 1: Figure S1.** Sec62 is involved in maintaining the stemness of CRC cells. **a**, Scatter plots of mRNA level of Sec62 versus mRNA levels of CD44, CD133, EPCAM, ITGB1, and BMI1 in colon adenocarcinoma from TCGA dataset were plotted. **b,** The control cells or Sec62 depleted cells were seeded in unattached 96-well plates and cultured for 7 day. The percentage of wells with spheres was evaluated and calculated with limiting dilution analysis (LDA) to determine the sphere-initiating cell frequency. Frequency and probability estimates were computed using the ELDA software. ***, *P* < 0.001. **c,** Sec62-overexpressed clones and the vector transcfected control cells were generated from DLD1 or HT29 cells. The protein levels of Sec62 in the stable clones were examined by Western blot. **d**, Western blot was performed as indicated using mice xenografts in Fig. 2g. **Figure S2.** Sec62 interacts with β-catenin. **a**, The amino acid sequences of Sec62 BCBL motif from different species were aligned. The critical conserved site of Sec62 is indicated by the red box. **b**, DA mutant lacking BCBL motif. **Figure S3.** Sec62 inhibits the APC-β-catenin interaction. **a**, DLD1 or HT29 cells were transfected as indicated. Then, cells were harvested and total proteins were subjected to Western blot using the indicated antibodies. **b**, RKO cells were transfected with the indicated plasmids. Co-immunoprecipitation assay was performed with anti-Flag antibody and the indicated proteins were evaluated by Western blot. **c**, DLD1 cells were transfected with the indicated plasmids. Co-immunoprecipitation assay was performed with anti-β-catenin antibody and the indicated proteins were evaluated by Western blot. **Figure S4.** Sec62 maintains the stemness of CRC by activating β-catenin signaling. **a,** Western blot analysis of Sec62, β-catenin, CD44 and c-Myc expression in 16 individual paired CRC tissues. **b**, Western blot was performed as indicated using mice xenografts in Fig. 7g. The relative CD44 or c-Myc protein levels were summarized (right). **Supplemental Table S1.** Reagents and antibodies. **Supplemental Table S2.** Correlation of Sec62 expression with clinicopathologic status in 102 cases of patients with colorectal cancer. **Supplemental Table S3.** shRNA sequence. **Supplemental Table S4.** Primer used for RT-qPCR.

## Data Availability

All data generated or analyzed during this study are included in this article and its supplementary files.

## Abbreviations

CRC: Colorectal cancer
CSC: Cancer stem cell
ER: Endoplasmic reticulum
DEGs: Differentially-expressed genes
AUC: Area under curve
BCBL: β-catenin binding like
ARM: Armadillo
AXIN1: Axis inhibition protein 1
APC: Adenomatous polyposis coli
METTL3: Methyltransferase-like protein 3
LGR5: Leucine-rich repeat-containing G-protein coupled receptor 5
IGF2BP1: Insulin-like growth factor 2 mRNA-binding protein 1
WT: Wild-type
RIP: RNA immunoprecipitation
IHC: Immunohistochemistry
RT-qPCR: Real-time quantitative PCR
