# Supplementary material for: Sec62 promotes stemness and chemoresistance of human colorectal cancer through activating Wnt/β-catenin pathway
Source: J Exp Clin Cancer Res. 2021 Apr 15;40:132. doi: 10.1186/s13046-021-01934-6 (PMC8051072; doi:10.1186/s13046-021-01934-6)
Supplement: Supplementary file 1 — Additional file 1: Figure S1. Sec62 is involved in maintaining the stemness of CRC cells. a, Scatter plots of mRNA level of Sec62 versus mRNA levels of CD44, CD133, EPCAM, ITGB1, and BMI1 in colon adenocarcinoma from TCGA dataset were plotted. b, The control cells or Sec62 depleted cells were seeded in unattached 96-well plates and cultured for 7 day. The percentage of wells with spheres was evaluated and calculated with limiting dilution analysis (LDA) to determine the sphere-initiating cell frequency. Frequency and probability estimates were computed using the ELDA software. ***, P < 0.001. c, Sec62-overexpressed clones and the vector transcfected control cells were generated from DLD1 or HT29 cells. The protein levels of Sec62 in the stable clones were examined by Western blot. d, Western blot was performed as indicated using mice xenografts in Fig. 2g. Figure S2. Sec62 interacts with β-catenin. a, The amino acid sequences of Sec62 BCBL motif from different species were aligned. The critical conserved site of Sec62 is indicated by the red box. b, DA mutant lacking BCBL motif. Figure S3. Sec62 inhibits the APC-β-catenin interaction. a, DLD1 or HT29 cells were transfected as indicated. Then, cells were harvested and total proteins were subjected to Western blot using the indicated antibodies. b, RKO cells were transfected with the indicated plasmids. Co-immunoprecipitation assay was performed with anti-Flag antibody and the indicated proteins were evaluated by Western blot. c, DLD1 cells were transfected with the indicated plasmids. Co-immunoprecipitation assay was performed with anti-β-catenin antibody and the indicated proteins were evaluated by Western blot. Figure S4. Sec62 maintains the stemness of CRC by activating β-catenin signaling. a, Western blot analysis of Sec62, β-catenin, CD44 and c-Myc expression in 16 individual paired CRC tissues. b, Western blot was performed as indicated using mice xenografts in Fig. 7g. The relative CD44 or c-Myc protein levels [file 13046_2021_1934_MOESM1_ESM.docx]

Supplemental Figures

**Figure S1**


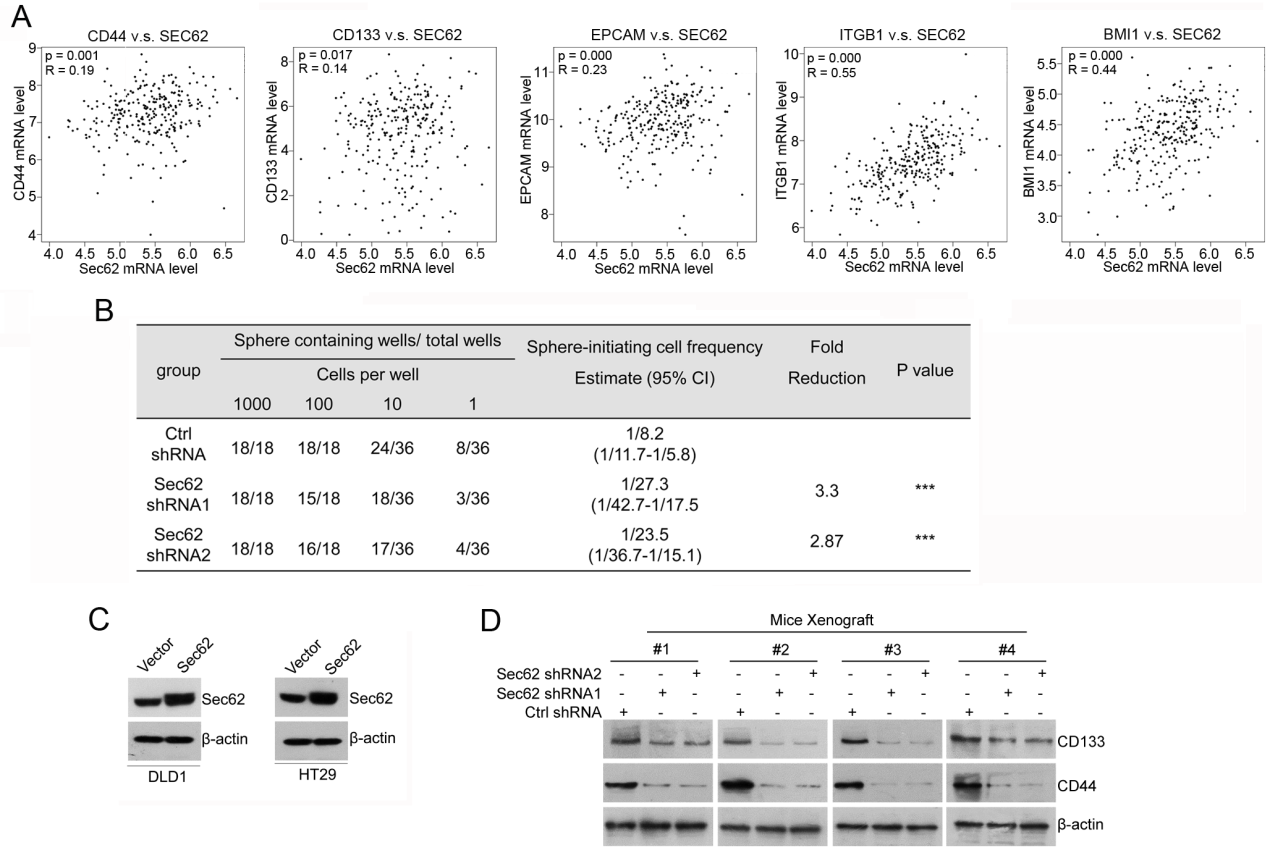
**Figure S1. Sec62 is involved in maintaining the stemness of CRC cells**. **a**, Scatter plots of mRNA level of Sec62 versus mRNA levels of CD44, CD133, EPCAM, ITGB1, and BMI1 in colon adenocarcinoma from TCGA dataset were plotted. **b,** The control cells or Sec62 depleted cells were seeded in unattached 96-well plates and cultured for 7 day. The percentage of wells with spheres was evaluated and calculated with limiting dilution analysis (LDA) to determine the sphere-initiating cell frequency. Frequency and probability estimates were computed using the ELDA software. ***, P < 0.001. **c,** Sec62-overexpressed clones and the vector transcfected control cells were generated from DLD1 or HT29 cells. The protein levels of Sec62 in the stable clones were examined by Western blot. **d**, Western blot was performed as indicated using mice xenografts in Figure 2g.

**Figure S2**


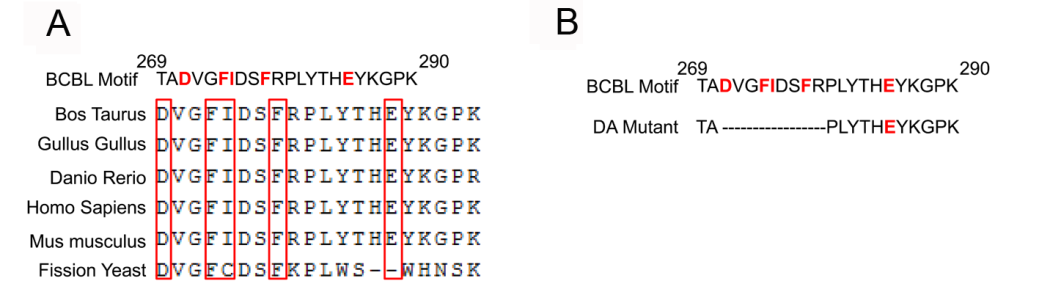


**Figure S2. Sec62 interacts with β-catenin**. **a**, The amino acid sequences of Sec62 BCBL motif from different species were aligned. The critical conserved site of Sec62 is indicated by the red box. **b**, DA mutant lacking BCBL motif.

**Figure S3**


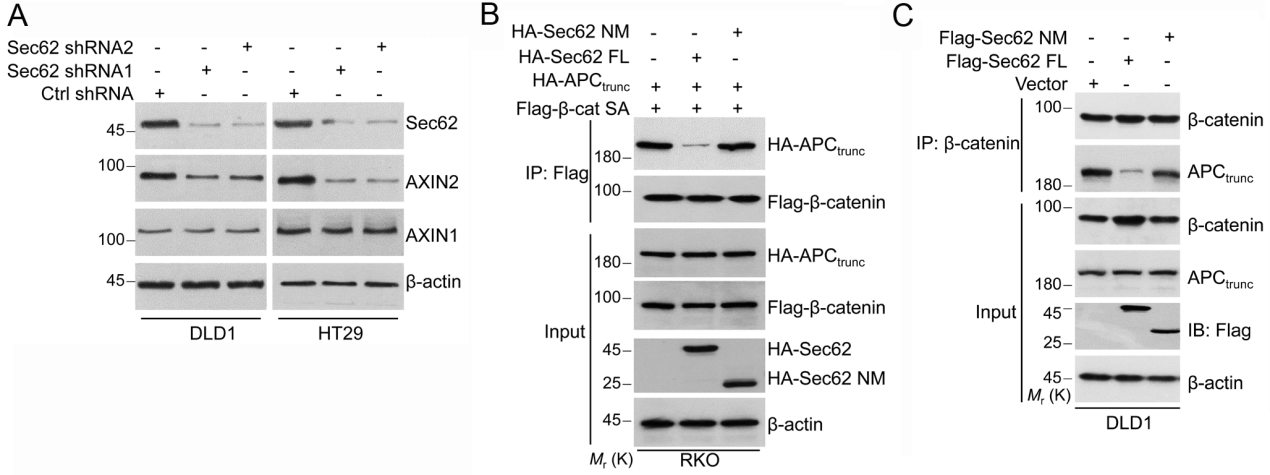


**Figure S3. Sec62 inhibits the APC-β-catenin interaction**. **a**, DLD1 or HT29 cells were transfected as indicated. Then, cells were harvested and total proteins were subjected to Western blot using the indicated antibodies. **b**, RKO cells were transfected with the indicated plasmids. Co-immunoprecipitation assay was performed with anti-Flag antibody and the indicated proteins were evaluated by Western blot. **c**, DLD1 cells were transfected with the indicated plasmids. Co-immunoprecipitation assay was performed with anti-β-catenin antibody and the indicated proteins were evaluated by Western blot.

**Figure S4**


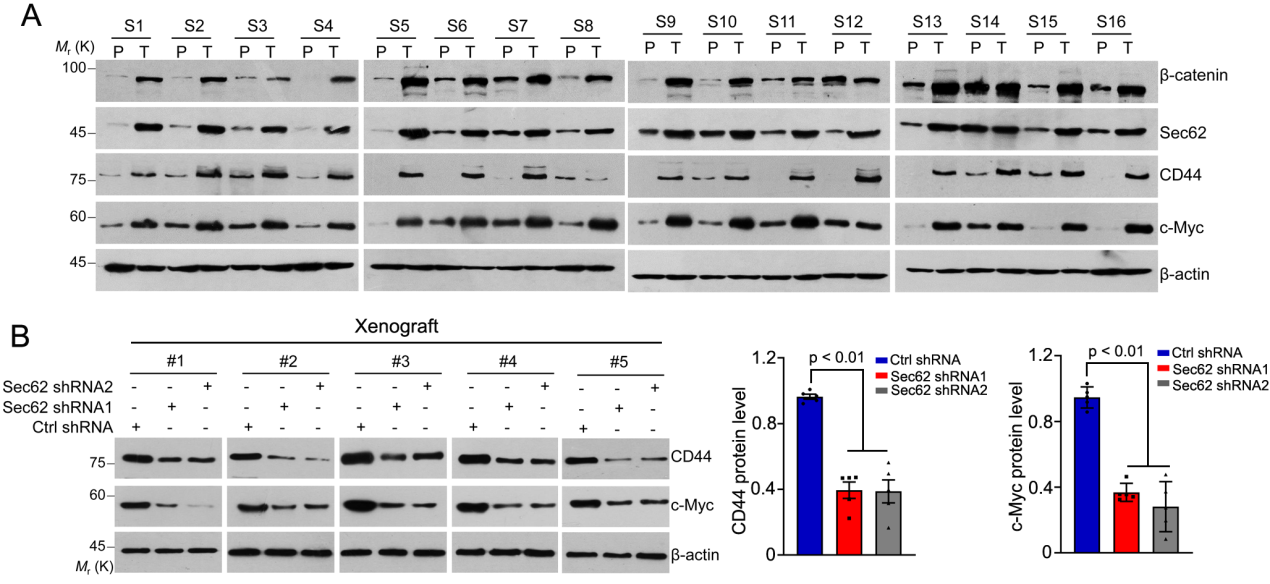


**Figure S4. Sec62 maintains the stemness of CRC by activating β-catenin signaling**. **a,** Western blot analysis of Sec62, β-catenin, CD44 and c-Myc expression in 16 individual paired CRC tissues. **b**, Western blot was performed as indicated using mice xenografts in Figure 7g. The relative CD44 or c-Myc protein levels were summarized (right).

**Supplemental Tables**

**Supplemental Table S1** Reagents and antibodies

| **Reagent** | **Company** | **Cat. No.** |
| --- | --- | --- |
| DMSO | Sigma -Aldrich | D2650 |
| Glutathione sepharose 4B | GE Healthcare, Piscataway, NJ | 17-0756-01 |
| iCRT14 | Santa Cruz Botech. | Sc-362746 |
| Wnt3a | Proteintech Group | A 17232 |
| LiCl | Sigma -Aldrich | L4408 |
| TWS119 | Abcam | Ab142075 |
| DMEM/F12 | Gibco | 11320082 |
| B27 Supplement | Gibco | 0080085SA |
| N2 Supplement | Gibco | 7502002 |
| insulin | Gibco | 51300044 |
| **Antibody** | **Company** | **Cat. No.** |
| anti-Sec62 | Abcam | Ab140644 |
| anti-Sec62 | Sigma -Aldrich | A96887 |
| anti-β-catenin | Cell Signaling Technology | 9582 |
| anti-β-catenin | Abclonal Tech., China | A11343 |
| anti-phos-β-catenin | Cell Signaling Technology | 9561 |
| anti-TCF4 | Cell Signaling Technology | 2565 |
| anti-APC | Cell Signaling Technology | 2504 |
| anti-AXIN1 | Cell Signaling Technology | 2087 |
| anti-cleaved caspase-3 | Cell Signaling Technology | 9661 |
| anti-CD133 | Abclonal Tech., China | A0219 |
| anti-CD44 | Abclonal Tech., China | A19020 |
| anti-Ub | Cell Signaling Technology | 3936 |
| anti-Flag | Sigma -Aldrich | F3165 |
| anti-Flag | TransGen Biotech | HT201 |
| anti-HA | TransGen Biotech | HT301 |
| anti-GFP | TransGen Biotech | HT801 |
| anti-GFP | Abclonal Tech., China | AE012 |
| anti-METTL3 | Abclonal Tech., China | A8370 |
| anti-m6A | Synaptic Systems | 202 111 |
| anti-IGF2BP1 | Proteintech Group | 22803-1-AP |
| anti-β-actin | Santa Cruz Botech. | sc-8432 |
| anti-β-actin | Abclonal Tech., China | AC026 |

**Supplemental Table S2** Correlation of Sec62 expression with clinicopathologic status in 102 cases of patients with colorectal cancer

| Variables | Total, n | Sec62 expression,^a^ n | | P value^b^ |
| --- | --- | --- | --- | --- |
|  |  | low | high |  |
| Gender |  |  |  | 0.071 |
| Male | 59 | 25 | 34 |  |
| Female | 43 | 26 | 17 |  |
| Age（year） |  |  |  | 0.963 |
| ≤ 65 | 47 | 25 | 22 |  |
| > 65 | 55 | 26 | 29 |  |
| Tumor location |  |  |  | 0.552 |
| Colon cancer | 49 | 26 | 23 |  |
| Rectal cancer | 53 | 25 | 28 |  |
| CEA（ng/mL） |  |  |  | 0.691 |
| ≤ 5 | 46 | 24 | 22 |  |
| > 5 | 56 | 27 | 29 |  |
| Tumour size （diameter, cm） |  |  |  | 0.843 |
| ≤ 4 | 53 | 27 | 26 |  |
| > 4 | 49 | 24 | 25 |  |
| Clinical Stage |  |  |  | **0.011** |
| Stage I | 13 | 7 | 6 |  |
| Stage II | 24 | 19 | 5 |  |
| Stage III | 43 | 18 | 25 |  |
| Stage IV | 22 | 7 | 15 |  |

^a^ According to the immunoreactive scores from immunohistochemistry of tissue microarray; Low, (0-5), high (6-12).

^b^ P values calculated using the chi-square test.

**Supplemental Table S3** shRNA sequence

| **Gene** | **sequence** |
| --- | --- |
| Sec62 shRNA#1  Sec62 shRNA#2  β-catenin shRNA  APC shRNA  METTL3 siRNA#1  METTL3 siRNA#2  IGF2BP1 siRNA#1  IGF2BP1 siRNA#2 | CCAGCACAGUAGUGGAAAU  GUACUUUCCACAGUUAAAU  CCATTGTTTGTGCAGCTGCTT  CCCAGTTTGTTTCTCAAGAAA  GGAGAUCCUAGAGCUAUUA  GCACAUCCUACUCUUGUAA  UUGAAUAGAAGCAGAAAAACAUU  UGCUAUUCUUCCUAAUCUAUAUC |

**Supplemental Table S4** Primer used for RT-qPCR

|  | **Primer Seuqence** |
| --- | --- |
| FLCN | F: TCTTCAGCATTGTCCGCCAG  R: AGTTGATGAGGTAGATCCGGTC |
| SPEF2 | F: CTTGGAGCCAACACTTAACCTT  R: GACGTTGCATGGTTTGCATCT |
| RPAH1 | F: ACAGTAAGCGTCAAATCACAGAA  R: GCAGCCTCATCCATACTCAAAG |
| SEC16B | F: GAAGGTGGTTATCGCAATCAGT  R: TGCCAGATATAAGGACTCCGTT |
| ZNF563 | F: AGACACGAAAAGTCTCACAGTG  R: CCTTGCATTTATTCGGCCCATC |
| PCDHGA4 | F: ATGATAACCCACCCAGTTTTGG  R: AGGGAGTTTACACCTACATCCG |
| SEC62 | F: GGCCAGCAGAAATGAGAGTAGG  R: AAGTGGTGCCTTCCTCCAGTT |
| GAPDH | F: TGCACCACCAACTGCTTAGC  R: GGCATGGACTGTGGTCATGAG |
| CD133 | F: CTGGGGCTGCTGTTTATTATTCTG  R: ACGCCTTGTCCTTGGTAGTGTTG |
| CD44 | F: TGGAGCAAACACAACCTCTG  R: TCCACTTGGCTTTCTGTCCT |
| LGR5 | F: CTCCCAGGTCTGGTGTGTTG  R: GAGGTCTAGGTAGGAGGTGAAG |
| CTNNB1 | F: CATCTACACAGTTTGATGCTGCT  R: GCAGTTTTGTCAGTTCAGGGA |
| AXIN2 | F: CAAGGGCCAGGTCACCAA  R: CCCCCAACCCATCTTCGT |
| MYC | F: GGCTCCTGGCAAAAGGTCA  R: CTGCGTAGTTGTGCTGATGT |
| SEC62 | F: TGTATTTTGAATATGAGCAAAAACTG  R: ACAAGAGTTTCCAAAGACTTGTAACTA |
| METTL3 | F: TTGTCTCCAACCTTCCGTAGT  R: CCAGATCAGAGAGGTGGTGTAG |
| IGF2BP1 | F: GCGGCCAGTTCTTGGTCAA  R: TTGGGCACCGAATGTTCAATC |
